# Inhibition of Cerebral High-Mobility Group Box 1 Protein Attenuates Multiple Organ Damage and Improves T Cell-Mediated Immunity in Septic Rats

**DOI:** 10.1155/2019/6197084

**Published:** 2019-02-10

**Authors:** Chao Ren, Xiu-hua Li, Yao Wu, Ning Dong, Ya-lin Tong, Yong-ming Yao

**Affiliations:** ^1^School of Medicine, Nankai University, Tianjin 300071, China; ^2^Trauma Research Center, Fourth Medical Center of the Chinese PLA General Hospital, Beijing 100048, China; ^3^Department of Burns and Plastic Surgery, The 181st Hospital of Chinese PLA, Guilin 541002, China; ^4^State Key Laboratory of Kidney Disease, The Chinese PLA General Hospital, Beijing 100853, China

## Abstract

Sepsis remains one of the leading causes of mortality in intensive care units, but there is a shortage of effective treatments. A dysregulated host immune response and multiple organ injury are major factors for the pathogenesis and progression of sepsis, which require specific mechanism and treatment. In the present study, we performed an intracerebroventricular (ICV) injection of BoxA, a specific antagonist of high-mobility group box 1 protein (HMGB1), in septic rats that were produced by cecal ligation and puncture surgery; we further assessed the functional changes of multiple organs and splenic T lymphocytes. We found that the inhibition of cerebral HMGB1 significantly alleviated multiple organ damage under septic exposure, including damage to the heart, liver, lungs, and kidneys; reversed the immune dysfunction of T cells; and increased the survival of septic rats. These data suggest that central HMGB1 might be a potential therapeutic target for septic challenge and that inhibition of brain HMGB1 can protect against multiple organ dysfunction induced by sepsis.

## 1. Introduction

Sepsis is a life-threatening condition that contributes to millions of deaths every year. The mortality caused by severe sepsis or septic shock remains high because the mechanisms remain unclear and a shortage of specific management methods remains despite a deep understanding and the extensive application of various treatments. Currently, aberrant immune response to infection is considered the major cause for sepsis, which is followed by multiple organ damage and even death [1]. The uncontrolled inflammatory response and refractory immune suppression are difficult issues when addressing the progression and prognosis of sepsis; these conditions are prone to cause either multiple organ dysfunction or recurrent infection without prompt interference [2]. Therefore, both anti-inflammatory response and immunomodulation are of great importance for the survival and prognosis of septic patients.

The neuroendocrine immune network is a major part of the immune-modulatory mechanism and is critically involved in the pathogenesis of the septic response. As an example, the cholinergic anti-inflammatory pathway (CAP) reportedly alleviates multiple organ injury and improves the survival of septic animals by downregulating the inflammatory response and effective immunomodulation [3, 4]. However, a disturbed response of CAP resulting from the dysfunction of brain nuclei, loss of nerve connection, or suppressed expression of the alpha7 nicotinic acetylcholine receptor is responsible for poor outcomes in septic settings [5]. Therefore, maintaining the functional homeostasis of the neuroendocrine immune network is important for the efficient treatment of sepsis. Brain injury has been identified as a major contributor for immunosuppression via inducing an abnormal response of the neuroendocrine immune axis [6, 7]. For instance, the vagus nerve has presented with increased tone and further brought about immunosuppression after traumatic brain injury (TBI), which might be partly due to the feedback of impaired brain nuclei [6]. Indeed, the brain has been reported to be the first organ that is subjected to exaggerated local inflammation under septic challenge and has been found to be prone to irreversible damage if timely and effective manipulation does not occur [8, 9]. In addition, the crosstalk of brain and peripheral organs was noted while addressing some critical states. Patients with severe TBI, as an example, commonly show elevated liver enzymes in early phases after injury [10]. This was identical to that seen with acute lung injury and acute respiratory distress syndrome, which are also commonly complicated by TBI as the result of a dysregulated immune response [11, 12]. Indeed, the interaction between the central nervous system and the peripheral immune response is a decisive factor in the outcomes of patients with critical illnesses, and this has been ascribed to distinct host immune suppression under severe brain damage [13]. Therefore, brain injury might act as part of a vicious cycle of anti-inflammation and immunomodulation that underlies the dysfunction of the neuroendocrine immune network, which should be addressed promptly.

Recently, excessive inflammatory mediator production has been documented to be a major cause for brain injury secondary to septic challenge. Tumour necrosis factor- (TNF-) *α*, for example, contributes to cerebral oedema and massive apoptosis of neurons under septic exposure; these did not occur under TNFR1 deficiency [14]. We also reported that an elevated expression of brain high-mobility group box 1 protein (HMGB1) results in brain tissue damage and a massive apoptosis of neural cells, followed by impaired memory and learning in septic mice [15]. Antagonism of cerebral HMGB1 significantly alleviated sepsis-induced brain injury, indicating that HMGB1 is a major contributor to sepsis-induced brain injury and represents a potential therapeutic target by virtue of its effective neuromodulation of the immune system [15]. However, the protective effect of the inhibition of cerebral HMGB1 on host immune response dysfunction and multiple organs remains unclear.

## 2. Materials and Methods

### 2.1. Animals

Sprague Dawley rats (male, weighing 250 ± 20 g) were purchased from Laboratory Animal Science of the Chinese Academy of Medical Sciences, Beijing, China. All experiments were performed according to the National Institutes of Health (NIH) Guidelines and were approved by the Scientific Investigation Board of the Chinese PLA General Hospital (no. SYXK2014-0018), Beijing, China.

### 2.2. Intracerebroventricular Cannulation and Injection

Rats were divided into three groups (according to a random number): the sham group, the sepsis group, and the sepsis with intracerebroventricular injection of BoxA group (sepsis+BoxA group). The intraventricular cannulation was performed by using a motorized rat stereotactic instrument (Stoelting Co., Wood Dale, IL), as reported previously [16]. The rats were placed on the apparatus after being anaesthetized by isoflurane inhalation (induction: 5%, maintenance: 3%). The skull was exposed after making a 2 cm sagittal incision, and the bregma was further located and set as coordinate zero (*x* = 0, *y* = 0, *z* = 0). A sterile catheter was then inserted into the left ventricle at predefined coordinates (*x* = −0.72 mm, *y* = 2.0 mm, *z* = 0), which was fixed using acrylic dental cement. The rats were allowed to rest for 7 days for recovery. The intraventricular injection was performed after successful anaesthesia. BoxA solution (1 *μ*g or 10 *μ*g in 5 *μ*l saline solution) was injected into the left lateral ventricle directly.

### 2.3. The Construction of Septic Rat Model

The rat model of sepsis was reproduced by cecal ligation and puncture (CLP) surgery. In brief, the fully exposed caecum was ligated at 75% of the distance from its distal pole to the base and further punctured with a 16-gauge needle. A bit of faeces were extruded to ensure the patency of punctured caecum. The caecum was returned, and the incision was closed. Then, rats underwent subcutaneous injection of 0.9% saline (5 ml per 100 g body weight) for resuscitation. In the sham group, rats were only subjected to cecal exposure.

### 2.4. Quantification of Serum Parameters for Organ Dysfunction

Blood samples were harvested and centrifuged to collect the serum which was used for analysing biochemical parameters via HITACHI 7600 biochemical analyser (Hitachi, Tokyo, Japan). The damage of the lungs was reflected by the ratio of wet-to-dry weight (W/D) and activity of myeloperoxidase (MPO). The wet weight of lung tissue was recorded immediately after its collection; then the dry weight and W/D were recorded after drying at 80°C for 24 h. The activity of pulmonary MPO was measured by enzyme-linked immunosorbent assay (ELISA, Hycult Biotech, Netherlands) after homogenization.

### 2.5. Preparation of Splenic CD4^+^ T Cells

Isolated spleens were dispersed through a 300-mesh sieve for producing single-cell suspension. The Ficoll-Paque density gradient centrifugation was then used for collecting mononuclear cells. The separation of splenic CD4^+^ T lymphocytes was performed through magnetic cell sorting system (Miltenyi Biotec, Bergisch Gladbach, Germany) following manufacturer's illustration.

### 2.6. Measurement of the Proliferative Activity of CD4^+^ T Cells

The cell counting kit-8 (CCK8, Dojindo Laboratories, Kumamoto, Japan) was used for assessing the proliferative activity of T cells in line with manufacturer's illustration. Briefly, the CD4^+^ T cells were cultured with concanavalin A (5 *μ*g/ml, Sigma, St. Louis, MO) for 68-72 h and further cocultured with CCK8 solution for 2 hours. Then, the optical density was recorded by ELISA plate reader (450 nm, Spectra MR, Dynex, Richfield, MN).

### 2.7. Enzyme-Linked Immunosorbent Assay

ELISA kits (Excell Inc., Shanghai, China) were used to measure the concentration of cytokines in culture supernatants following manufacturer's protocol, including interleukin- (IL-) 2, IL-4, and interferon- (IFN-) *γ*. The results were measured at 450 nm using the ELISA plate reader.

### 2.8. Quantitative Real-Time Polymerase Chain Reaction (qPCR)

The qPCR was applied for measuring mRNA expressions of IL-2 and IL-4 as well as IFN-*γ*. Total RNA was extracted using Trizol reagent (Invitrogen, California) in accordance with manufacturer's protocols. The synthesis and preparation of cDNA were conducted via reverse transcription system (Promega, Madison, WI). Then, the cDNA was quantified and analysed using SYBR Green PCR Master Mix and the following primers: rat IL-2, 5′CAGCGTGTGTTGGATTTGAC3′ (forward) and 5′TGATGCTTTGACAGATGG CTA3′ (reverse); rat IL-4, 5′AACAAGTCTGGGGTTCTCGG3′ (forward) and 5′TGTTGTGAGCGTGGACTCAT3′ (reverse); and rat IFN-*γ*, 5′AGGTGAACAACCCACAGAT3′ (forward) and 5′CTTCTTATTGGCACACTCTCTAC3′ (reverse). Gene expression was normalized to that of *β*-actin and was evaluated using the value of 2^−dCt^.

### 2.9. Statistical Analysis

SPSS 19.0 software was used for data analysis, and the results are presented as the means ± standard deviation (SD). Differences among multiple groups were evaluated by one-way analysis of variance (ANOVA). The Student *t*-test was used for assessing significant intergroup differences. Differences of survival rates are shown using Kaplan-Meier survival curves and were further analysed using the log rank test. *P* values < 0.05 were considered significant.

## 3. Results

### 3.1. Central HMGB1 Inhibition Dose-Dependently Ameliorated Sepsis-Induced Multiple Organ Dysfunction

**Figure 1:**
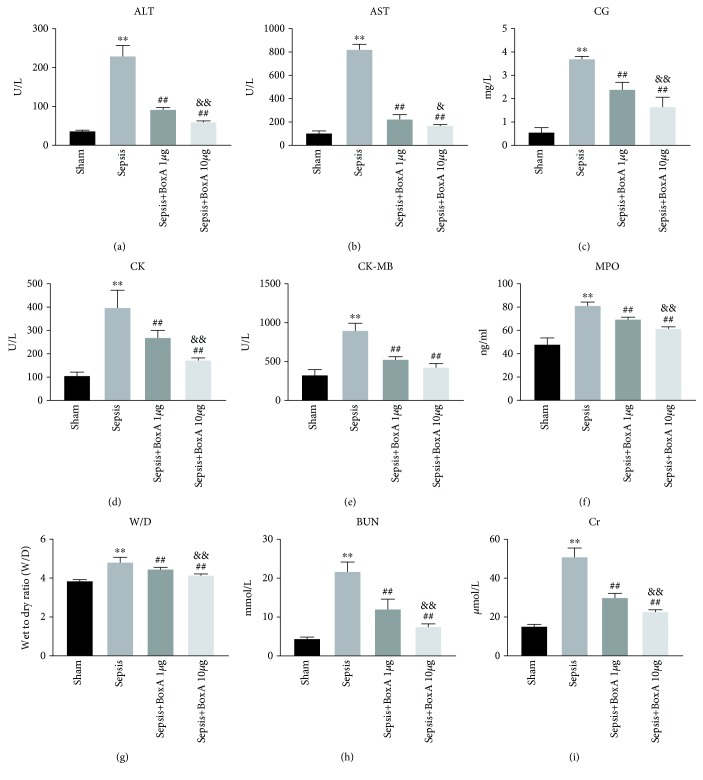
Intracerebroventricular (ICV) injection of BoxA ameliorated multiple organ damage under sepsis exposure. (a–e, h, i) Serum biochemical parameters, including creatine kinase (CK), CK-MB, aspartate aminotransferase (AST), alanine aminotransferase (ALT), cholylglycine (CG), blood urea nitrogen (BUN), and creatinine (Cr), were measured using a HITACHI 7600 biochemical analyser as these parameters reflect multiple organ dysfunction. Serum samples were collected at 24 h after cecal ligation and puncture (CLP) surgery. BoxA solution (1 *μ*g or 10 *μ*g) was injected into the left lateral ventricle immediately after operation. (f, g) The wet-to-dry ratio (W/D) and myeloperoxidase (MPO) activity of pulmonary tissues were quantified by weighing and using an ELISA kit, respectively. Lungs were harvested and weighed at 24 h after CLP surgery. (Each bar represents the mean ± SD of three independent experiments, *n* = 6; ^∗∗^*P* < 0.01 vs. the sham group; ^##^*P* < 0.01 vs. the sepsis group; ^&^*P* < 0.05 vs. the sepsis plus 1 *μ*g BoxA group; ^&&^*P* < 0.01 vs. the sepsis plus 1 *μ*g BoxA group.)

The function of multiple organs was assessed based on serum biochemical parameters at 24 h post CLP surgery. As shown in Figures 1(a)–1(c), indicators for liver injury, including aminotransferase (AST), alanine aminotransferase (ALT), and cholylglycine (CG), all presented higher levels in the sepsis group than in the sham group. Concurrently, damage of the heart, kidneys, and lungs was also noted in the sepsis group, as evidenced by elevated concentrations of serum CK, CK-MB, BUN, and Cr as well as increased activity of MPO and W/D ratio in pulmonary tissues when compared with those of the sham group (Figures 1(d)–1(i)). Intracerebroventricular (ICV) injection of BoxA significantly ameliorated multiple organ damage, as shown by decreased serum levels of ALT, AST, CG, CK, CK-MB, BUN, and Cr, as well as reduced MPO activity and W/D ratio of pulmonary tissues when compared with those of the sepsis group. Moreover, the beneficial effects of ICV injection of BoxA on multiple organ dysfunction were dose-dependent (Figure 1).

### 3.2. Intraventricular Injection of BoxA Attenuated Sepsis-Induced Multiple Organ Injury in Time-Dependent Ways

**Figure 2:**
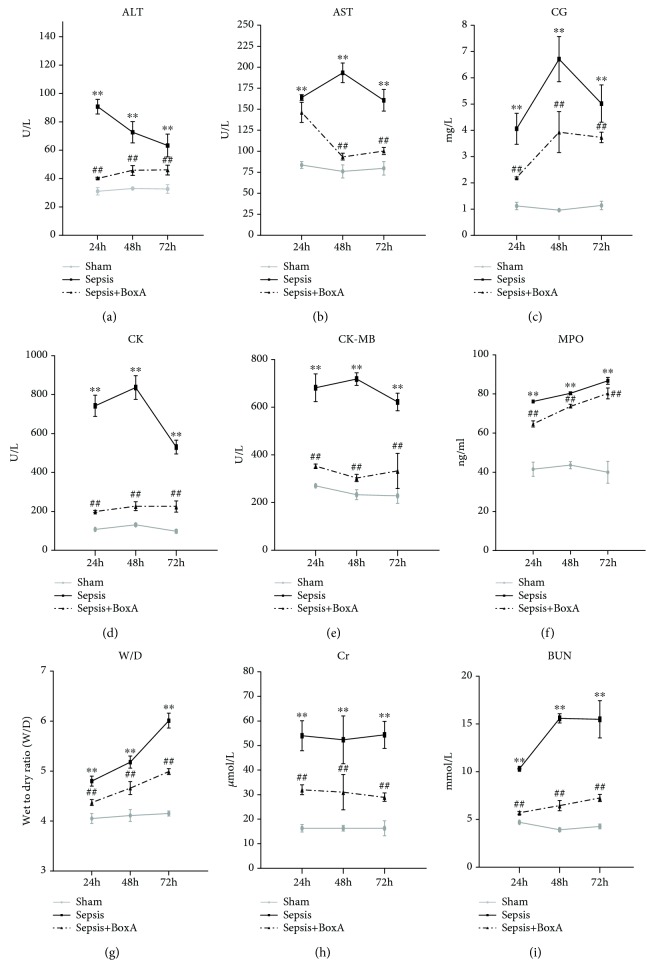
Time-dependent effects of an ICV injection of BoxA on multiple organ damage induced during sepsis. (a–e, h, i) Serum biochemical parameters, including creatine kinase (CK), CK-MB, aspartate aminotransferase (AST), alanine aminotransferase (ALT), cholylglycine (CG), blood urea nitrogen (BUN), and creatinine (Cr), were determined using a HITACHI 7600 biochemical analyser as these parameters reflect multiple organ dysfunction. Serum samples were collected at 24 h, 48 h, and 72 h after cecal ligation and puncture (CLP) surgery. A dose of 10 *μ*g BoxA was injected into the left lateral ventricle at 0 h, 24 h, and 48 h after CLP surgery. (f, g) The W/D and MPO activities of lung tissues were quantified by weighing and the use of an ELISA kit, respectively. Lungs were harvested and weighed at 24 h, 48 h, and 72 h after CLP surgery. (Each point represents the mean ± SD of three independent experiments, *n* = 6; ^∗∗^*P* < 0.01 vs. the sham group; ^##^*P* < 0.01 vs. the sepsis group.)

To deepen our understanding of the role of brain HMGB1 inhibition in the resolution of sepsis, the time-dependent effects of BoxA injection (10 *μ*g based on dose-dependent results) were further evaluated at 24 h, 48 h, and 72 h post CLP surgery. In the sepsis group, serum markers for the heart, liver, and kidneys as well as indicators for lung injury were all found to have significantly increased during the course of sepsis in comparison with the sham group. The administration of BoxA significantly ameliorated sepsis-induced multiple organ damage at 24 h, 48 h, and 72 h after sepsis initiation as evidenced by the declining levels of serum biomarkers for organ dysfunction as well as decreasing lung oedema and MPO content (Figure 2).

### 3.3. The Dose-Dependent Effects of ICV Injection of BoxA on the Proliferation, Secretion, and Differentiation of Splenic CD4^+^ T Cells

**Figure 3:**
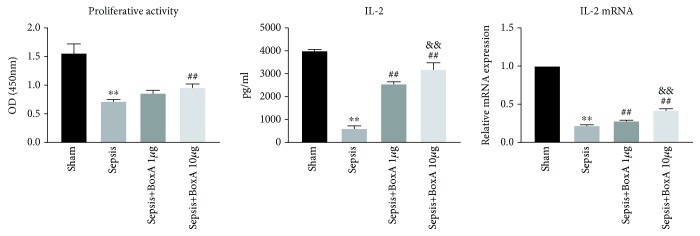
Dose-dependent effects of an ICV injection of BoxA on the proliferation and secretion of T lymphocytes in sepsis. The proliferative activity of T cells was assessed using a CCK8 kit after culture with concanavalin A for 68-72 h and was further recorded at 450 nm using an ELISA plate reader. The protein and mRNA expression levels of interleukin-2 (IL-2) were measured using an ELISA kit and the quantitative real-time polymerase chain reaction (qPCR), respectively. (Each bar represents the mean ± SD of three independent experiments; the qPCR data were analysed using the value of 2^−dCt^, and the data are presented as the ratio of the result to that in the sham group *n* = 6; ^∗∗^*P* < 0.01 vs. the sham group; ^##^*P* < 0.01 vs. the sepsis group; ^&&^*P* < 0.01 vs. the sepsis plus 1 *μ*g BoxA group.)

**Figure 4:**
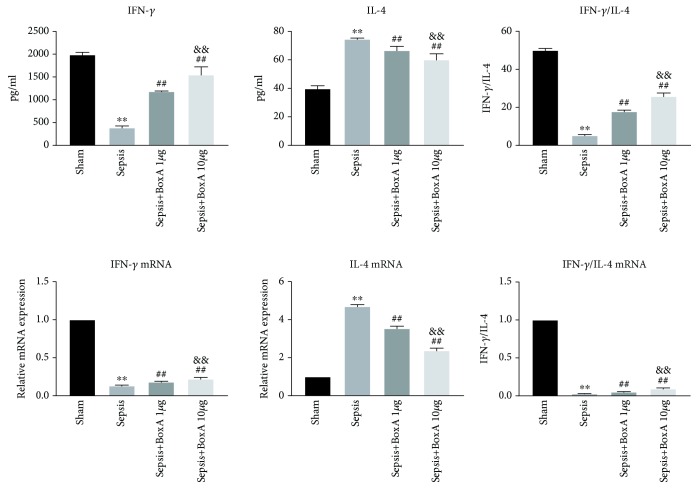
Dose-dependent effects of an ICV injection of BoxA on the differentiation of T cells in sepsis. The differentiation of T lymphocytes was analysed based on the ratio of interferon-*γ* (IFN-*γ*) to interleukin-4 (IL-4) (IFN-*γ*/IL-4). Levels of IFN-*γ* and IL-4 protein and mRNA were quantified by ELISA and qPCR, respectively. (Each bar represents the mean ± SD of three independent experiments; the qPCR data were analysed using the value of 2^−dCt^, and the data are presented as the ratio of the result to that in sham group, *n* = 6; ^∗∗^*P* < 0.01 vs. the sham group; ^##^*P* < 0.01 vs. the sepsis group; ^&&^*P* < 0.01 vs. the sepsis plus 1 *μ*g BoxA group.)

The functional status of CD4^+^ T cells is essential for the immune response in the setting of sepsis. The proliferation of CD4^+^ T cells was inhibited in the sepsis group when compared to the sham group (Figure 3). IL-2 expression and release in CD4^+^ T cells was reduced under sepsis exposure, and this was reversed by inhibiting central HMGB1, especially at the dose of 10 *μ*g BoxA. The proliferative arrest of CD4^+^ T cells that were compromised by sepsis was also improved by the central administration of 10 *μ*g BoxA, but no significant alteration was observed at a dose of 1 *μ*g BoxA. The differentiation of CD4^+^ T cells was analysed based on the ratio of IFN-*γ* to IL-4. As shown in Figure 4, protein and mRNA expression of IFN-*γ* was significantly lower in the sepsis group than that in the sham group, while IL-4 expression was enhanced under septic challenge, along with lower ratio of IFN-*γ* to IL-4 when compared to that in the sham controls. The discrepant expression of IFN-*γ* and IL-4 exhibited the dominant differentiation of Th2 cells. However, the ICV injection of BoxA significantly restored the aberrant differentiation of CD4^+^ T cells by dose-dependently elevating IFN-*γ* expression and reducing IL-4 release (Figure 4).

### 3.4. The Time-Dependent Effects of BoxA on the Function of CD4^+^ T Cells

**Figure 5:**
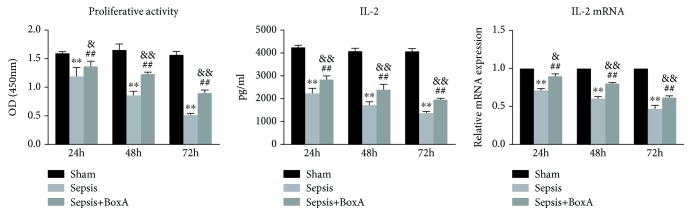
Time-dependent effects of an ICV injection of BoxA on the proliferation and secretion of T lymphocytes under septic challenge. The proliferative activity of T cells was assessed using a CCK8 kit after culture with concanavalin A for 68-72 h and was further recorded at 450 nm using an ELISA plate reader. Interleukin-2 (IL-2) protein and mRNA levels were measured using an ELISA kit and qPCR, respectively. (Each bar represents the mean ± SD of three independent experiments; the qPCR data were analysed using the value of 2^−dCt^, and the data are presented as the ratio of the result to that in the sham group, *n* = 6; ^∗∗^*P* < 0.01 vs. the sham group; ^##^*P* < 0.01 vs. the sepsis group, ^&^*P* < 0.05 vs. the sham group, ^&&^*P* < 0.01 vs. the sham group.)

**Figure 6:**
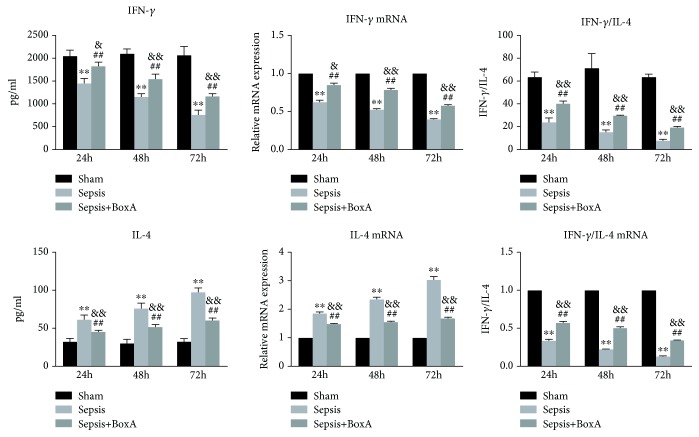
Time-dependent effects of an ICV injection of BoxA on the differentiation of T cells during the course of sepsis. The differentiation of T lymphocytes was analysed based on the ratio IFN-*γ*/IL-4. Interferon-*γ* (IFN-*γ*) and interleukin-4 (IL-4) protein and mRNA levels were quantified by ELISA and qPCR, respectively. (Each bar represents the mean ± SD of three independent experiments; the qPCR data were analysed using the value of 2^−dCt^, and the data are presented as the ratio of the result to that in the sham group, *n* = 6; ^∗∗^*P* < 0.01 vs. the sham group; ^##^*P* < 0.01 vs. the sepsis group, ^&^*P* < 0.05 vs. the sham group, ^&&^*P* < 0.01 vs. the sham group.)

As shown in Figure 5, both the proliferative activity and IL-2 expression of CD4^+^ T cells were downregulated at 24 h, 48 h, and 72 h after CLP. The administration of BoxA reversed the proliferative arrest of CD4^+^ T cells during the course of sepsis and was accompanied by the elevated expression of IL-2 (both protein and mRNA) when compared with the sepsis group. However, the injection of BoxA did not alter the proliferative activity and IL-2 expression in the sham group range, as significant differences were also noted between the sepsis with BoxA group and the sham group. The expression of IFN-*γ* showed a time-dependent decline during the progression of sepsis, as seen in Figure 6. However, the protein and mRNA levels of IL-4 gradually increased with the development of sepsis, and higher levels were noted at 72 h than at 24 h and 48 h post CLP surgery. The CD4^+^ T cells showed the polarization of Th2 cells with a lower ratio of IFN-*γ*/IL-4. Central treatment with BoxA improved the abnormal response of CD4^+^ T cells in sepsis by enhancing the expression of IFN-*γ* and decreasing that of IL-4, following an increase in the ratio IFN-*γ*/IL-4. The response of CD4^+^ T cells in the sepsis with BoxA group remained below the level in the sham group, as shown in Figure 6.

### 3.5. The Effect of Central HMGB1 Inhibition on the Survival of Septic Rats

**Figure 7:**
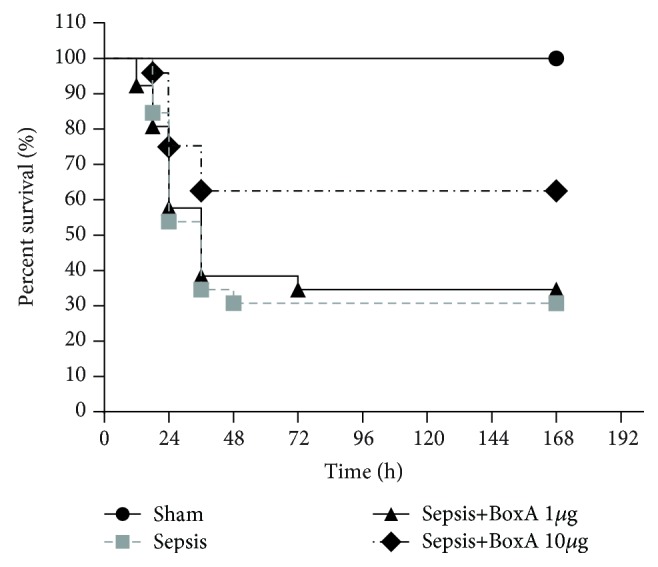
Effects of an ICV injection of BoxA on the survival rates of septic rats. The survival rates of septic rats were recorded at 12 h, 24 h, 36 h, 48 h, 72 h, 96 h, 120 h, 144 h, and 168 h after the onset of sepsis. Central administration of BoxA at a dose of 10 *μ*g significantly improved the survival of septic rats in comparison with rats in the sepsis group (the sepsis+10 *μ*g BoxA group vs. the sepsis group: 62% vs. 30%, *P* < 0.05, *n* = 26). No significant improvement was found in survival rates when using a low dosage of BoxA injection in comparison with that of the sepsis group (the sepsis+1 *μ*g BoxA group vs. the sepsis group: 34% vs. 30%, *P* > 0.05, *n* = 26).

The survival rates were recorded at 12 h, 24 h, 36 h, 48 h, 72 h, 96 h, 120 h, 144 h, and 168 h post CLP surgery (Figure 7). In the sepsis group, only 30% of the rats survived from sepsis within 168 h, lower than the percentage in the sham group (the sepsis group vs. the sham group: 30% vs. 100%, *P* < 0.05, *n* = 26). The central administration of BoxA at a dose of 10 *μ*g significantly improved the survival rates when compared with the sepsis group (the sepsis+BoxA 10 *μ*g group vs. the sepsis group: 62% vs. 30%, *P* < 0.05, *n* = 26). Nevertheless, no significant improvement was noticed in survival rates with the low dosage of BoxA injection in comparison with the sepsis group (the sepsis+BoxA 1 *μ*g group vs. the sepsis group: 34% vs. 30%, *P* > 0.05, *n* = 26).

## 4. Discussion

HMGB1 has been identified as an essential therapeutic target for sepsis due to its efficacy in driving an uncontrolled inflammatory response and poor outcomes [17, 18]. Inhibiting the release of HMGB1 protects against severe sepsis, as shown by the alleviation of multiple organ dysfunction and the improved survival of septic animals [19]. HMGB1 overproduction has been documented to be responsible for organ injury in various diseases. In brain injury caused by subarachnoid haemorrhage, for example, HMGB1 presented with quick translocation and release at 2 hours after injury, resulting in brain damage through the triggering of local inflammation, which was eliminated by the administration of HMGB1 inhibitors [20]. Additionally, the persistent injection of HMGB1 into naïve mice induced a significant impairment in memory and learning, indicating that extracellular HMGB1 might be an independent factor in central nervous system dysfunction through exaggerating the local inflammatory response [21]. In the setting of septic challenge, the production of central HMGB1 has been identified as a major contributor to cognitive dysfunction, as HMGB1 antagonism significantly alleviated brain injury and offered improvement in neurological disorders, indicating that the enhanced production of HMGB1 should be noted and addressed with prompt interference to protect the central nervous system against severe sepsis [15, 21]. As mentioned above, functional homeostasis of the brain was shown to be of great importance in effective immune modulation as a major component of the neuroendocrine immune network but with an intricate mechanism and ambiguous targets [6, 7]. Therefore, we hypothesized that central HMGB1 might be a potential therapeutic target underlying the aberrant immune system response and poor outcomes of septic animals by inducing dysfunction of the central nervous system.

In the current study, we found that the ICV injection of BoxA, a specific antagonist for HMGB1, significantly and dose-dependently alleviated multiple organ damage, including damage to the heart, liver, lungs, and kidneys. To our knowledge, this is the first report showing the beneficial effect of the central antagonism of HMGB1 in mitigating the multiple organ dysfunction induced by sepsis underlying the connection between brain and peripheral organs. The interplay between the central nervous system and peripheral organs has also been noted in other critical conditions. Brain dysfunction is commonly complicated by damage to the peripheral organs for reasons including inflammatory insults, dysbolism, and ischaemic injury as well as stress from toxic metabolites, as reported previously [22–24]. Likewise, peripheral organs were also subjected to functional disturbance under acute brain injury. For instance, myocardial dysfunction presents frequently during subarachnoid haemorrhage, as documented by Min et al. [25]. Many other organs, such as the lungs and kidneys, also showed dysfunction under exposure to severe brain injury, possibly due to an abnormal response of the peripheral immune system [10, 11, 13]. Given the two crucial factors for the prognosis of septic patients, namely, a dysregulated immune response and multiple organ damage, it will be more important to clarify the crosstalk between the brain and peripheral organs underlying potential therapeutic targets for neuromodulation as the brain has been reported to be the first organ affected by septic challenge. The present study focused on the inhibition of cerebral HMGB1 in alleviating multiple organ damage by virtue of the protective effects on sepsis-induced brain injury, thus highlighting the therapeutic value of central HMGB1 for septic response [15, 21].

We further assessed the functional changes of splenic T lymphocytes after the induction of sepsis. The proliferative activity of T cells was suppressed following abdominal sepsis together with a decrease in the expression of IL-2. Meanwhile, the differentiation of T lymphocytes was dysregulated under sepsis exposure as shown by the polarization of Th2 cells due to the decreased expression of IFN-*γ* but enhanced production of IL-4, indicating the outward signs of immunosuppression. The inhibition of cerebral HMGB1 via an ICV injection of BoxA dose-dependently reversed the proliferative arrest of T cells; this was accompanied by an enhanced expression of IL-2 and an improved differentiation status. The protective role of the ICV injection of BoxA was also noted to vary over time; however, the rescue might be partial as the indicators for the BoxA-treated group were not in the range of those in the sham group. These results provide direct evidence that the inhibition of central HMGB1 has advantages for maintaining the functional homeostasis of the peripheral immune response. In fact, brain HMGB1 was confirmed to be a crucial mediator for the peripheral immune response under acute brain lesions [26]. In the setting of sepsis involving both an uncontrolled system inflammatory response and refractory immunosuppression, the neutralization of central HMGB1 activity might be an effective target for breaking the vicious cycle of disturbed neuromodulation caused by brain injury. Recently, multiple inflammatory reflexes have been shown to account for the interplay between central inflammatory mediators and the peripheral immune system. For instance, the cholinergic anti-inflammatory pathway is one of the most studied mechanisms due to its potent anti-inflammation and immunomodulatory effects in inflammatory diseases [27–29]. The activation of the cholinergic system protects against system inflammatory insults and multiple organ dysfunction in the septic state, resulting in an increased survival of septic animals [30]. Moreover, the disturbed activation of the brain cholinergic system has been shown to be unfavourable for immune function after the onset of sepsis (this has been ascribed to an exaggerated response of both local and systemic inflammation), implying that neuroinflammation is a major cause of the dysfunction of brain cholinergic nuclei [5, 31]. Thus, the reversal of the disturbed response of T lymphocytes due to the ICV injection of BoxA might be partly due to the great capacity of HMGB1 for triggering local inflammation, as mentioned above [20]. In addition to assessing multiple organ damage and the immune function of T lymphocytes, we further evaluated the survival rates within 7 days post CLP surgery and found that the inhibition of cerebral HMGB1 significantly improved the survival of septic rats. These data strengthened the insights into the protective role of central HMGB1 antagonism against severe sepsis in very precise and economical ways. It should be noted that the dose of BoxA used for the ICV injection was too low to work in a systemic way, as there is a considerable difference between the doses needed for central and peripheral administration for immune modulation [32]. Therefore, an increased release of cerebral HMGB1 should be recognized and promptly addressed as it underlies the protective effects of the intact neuroendocrine immune network against sepsis.

The present study shares a novel insight into the protective effect of the inhibition of cerebral HMGB1 activity in alleviating multiple organ damage and reversing the abnormal immunity of T lymphocytes, resulting in an improved survival of septic rats. The results might be helpful for understanding the pathogenesis and progression of sepsis and for further exploring the therapeutic targets for septic challenge. However, we have only described the advantage of the antagonism of central HMGB1 in sepsis; the mechanism underlying the crosstalk between the brain and peripheral organs remains to be clarified. Both neural and humoral pathways were responsible for the immune modulation of cerebral HMGB1 by inducing an inflammatory reflex and passing through the blood-brain barrier with redox modification [33]. Weber and colleagues found that the HMGB1-RAGE axis was responsible for lung dysfunction induced by traumatic brain injury but without illustrating the effects of cerebral HMGB1 [34]. Therefore, both the brain and peripheral immune system should be taken into consideration while exploring the specific effects and mechanism associated with HMGB1 in further research. However, the problem remains difficult because the brain-immune signalling has not been clarified. Some neural pathways have been identified as critically involved in immunomodulation during sepsis and show great potential for maintaining immune response homeostasis and improving survival. Therefore, understanding the relationship between cerebral HMGB1 and the activity of these pathways that cover both the central and peripheral systems will undoubtedly strengthen our knowledge of its role in the development of sepsis and provide more targets for treating septic cases.

## 5. Conclusions

In summary, these results suggest a novel role of cerebral HMGB1 in the pathogenesis and progression of the septic response. The inhibition of brain HMGB1 strongly protects against abdominal sepsis by alleviating multiple organ damage and reversing the aberrant response of T lymphocytes, resulting in an increased survival of septic rats.

## Data Availability

The data used to support the findings of this study are included within the article.
